# The role of autophagy in tick-endosymbiont interactions: insights from *Ixodes scapularis* and *Rickettsia buchneri*

**DOI:** 10.1128/spectrum.01086-23

**Published:** 2023-12-01

**Authors:** Xin-Ru Wang, Benjamin Cull, Jonathan D. Oliver, Timothy J. Kurtti, Ulrike G. Munderloh

**Affiliations:** 1 Department of Entomology, University of Minnesota, St. Paul, Minnesota, USA; 2 SUNY Center for Vector-Borne Diseases, Upstate Medical University, Syracuse, New York, USA; 3 Institute for Global Health and Translational Sciences, Upstate Medical University, Syracuse, New York, USA; 4 Department of Microbiology and Immunology, Upstate Medical University, Syracuse, New York, USA; 5 Division of Environmental Health Sciences, School of Public Health, University of Minnesota, Minneapolis, Minnesota, USA; National Institutes of Health, Rockville, Maryland, USA

**Keywords:** *Rickettsia buchneri*, tick-endosymbiont interactions, autophagy, apoptosis

## Abstract

Approximately 80 identified tick species are recognized as vectors and impact global public health by transmitting a wide range of pathogens; however, little is known about the interactions of ticks with the microbiome that they harbor, let alone their bacterial symbionts. In this study, we used the black-legged tick, *Ixodes scapularis* Say (Acari: Ixodidae), a vector of seven human pathogens in the United States, and utilized *Rickettsia buchneri sensu stricto* ISO7^T^ (Rickettsiales: Rickettsiaceae), a spotted fever group (SFG) rickettsia that is an endosymbiont of *I. scapularis,* to investigate the role of autophagy in symbiont and tick interactions. We found that the expression profile of most autophagy family member proteins (ATGs) is down-regulated after *R. buchneri* infection in tick cell cultures. The autophagic process was observed by assessment of autophagosome formation and maturation *in vitro* (tick cell culture) and *in vivo* (tick ovary) in the presence of *R. buchneri*, whereas apoptosis was not induced. We further showed that *R. buchneri* infection triggered the accumulation of plasma membranes within cells. Suppressing autophagy via *Atg8* siRNA interference inhibited intracellular rickettsial replication. This research indicates that autophagy regulation is important for the maintenance of *R. buchneri* in its *I. scapularis* tick host and provides more clues to solve the tick-symbiont interaction puzzle.

**IMPORTANCE:**

Ticks are second only to mosquitoes in their importance as vectors of disease agents; however, tick-borne diseases (TBDs) account for the majority of all vector-borne disease cases in the United States (approximately 76.5%), according to Centers for Disease Control and Prevention reports. Newly discovered tick species and their associated disease-causing pathogens, and anthropogenic and demographic factors also contribute to the emergence and re-emergence of TBDs. Thus, incorporating different tick control approaches based on a thorough knowledge of tick biology has great potential to prevent and eliminate TBDs in the future. Here we demonstrate that replication of a transovarially transmitted rickettsial endosymbiont depends on the tick’s autophagy machinery but not on apoptosis. Our findings improve our understanding of the role of symbionts in tick biology and the potential to discover tick control approaches to prevent or manage TBDs.

## INTRODUCTION

Programmed cell death, as a highly conserved and crucial homeostatic mechanism from eukaryotic multicellular organisms to bacteria, is a genetically regulated process of cell suicide events, including apoptosis (type I), autophagy (type II), and necrosis (type III) (1, 2). Unlike apoptosis, autophagy is typically recognized as a survival strategy in development and response to nutrient starvation and eliminating unnecessary or dysfunctional components (3), including intracellular pathogens. The classical functions of autophagy and apoptosis have been well demonstrated in certain models (*Caenorhabditis elegans*, *Drosophila melanogaster*, and *Saccharomyces cerevisiae*) and mammals; yet, this does not include ticks, in which these processes are poorly understood (4 – 7). A series of cellular morphological and biochemical features can be used as markers for autophagy and apoptosis, and these are also applicable to studies in ticks. For instance, visualization of the autophagosome (large cytosolic double‐membrane vesicle) is a hallmark of the initiation of the autophagy process, which is controlled by a series of autophagy-related proteins (ATG) beginning with the initial sequestering of the phagophore (a cup-shaped membrane structure). Subsequently, fusion with the lysosome forms autolysosomes in which cytosol and organelles are degraded to recycle the macromolecules (8). Cell shrinkage, chromatin condensation, nuclear and chromosomal DNA fragmentation, and caspase cleavage, can be used to measure apoptosis, being the major characteristics of the apoptotic process (9). Current studies are focused on two main aspects of tick autophagy and apoptosis: (i) contribution to tick development, such as initiation of salivary gland degeneration through apoptosis following completion of a blood meal, embryonic patterning via autophagy during development, and autophagy induction in response to amino acid starvation; (ii) evolution of interactions between ticks and their transmitted pathogens, such as supporting successful infection/replication (*in vitro* studies) and colonization/persistence (*in vivo* studies) of some bacteria, or regulating pathogen activity as part of the innate surveillance mechanism (10 – 18). Our understanding of all the pathways in which autophagy plays a role is still far from complete, in part due to the broad range of pathogens transmitted by tick species. Moreover, only certain tick species and the pathogens they harbor have been studied with a multi-omics approach, which limits advances in the field in general. Interestingly, although non-pathogenic microbes make up the majority of the complex microbial communities within ticks, their impact on tick development and TBDs has received less attention, let alone how they might interfere with autophagy (19).

Thus, we utilized a medically important tick species, the black-legged tick, *Ixodes scapularis* Say (Acari: Ixodidae), to investigate the effect of non-pathogenic bacteria on tick autophagy. As a three-host tick with four life stages, *I. scapularis* feeds on a large variety of host animals and is an important vector for seven human pathogens in the United States, including *Borrelia burgdorferi* and *Borrelia mayonii* (the causative agents of Lyme disease), *Borrelia miyamotoi* (the causative agent of *B. miyamotoi* disease), *Anaplasma phagocytophilum* (the causative agent of human anaplasmosis), *Babesia microti* (a causative agent of human babesiosis), *Ehrlichia muris eauclairensis* (a causative agent of ehrlichiosis), and Powassan virus (the causative agent of Powassan encephalitis) (20 – 25). *Rickettsia buchneri* (formerly rickettsial endosymbiont of *I. scapularis*, or REIS) have been shown to be present in *I. scapularis* throughout the tick’s life cycle and to be highly prevalent in tick populations (26, 27), consistent with a role as primary symbiotic bacteria. The species status of *R. buchneri sensu stricto* using the ISO-7^T^ isolate was recently evaluated using digital DNA: DNA hybridization and a genome-to-genome distance calculator program (28). The analysis suggested that *R. buchneri* may be a subspecies of *Rickettsia tamurae* (AT-1T) (29), but this should be confirmed using additional studies. *R. buchneri sensu stricto* are transovarially transmitted endosymbionts that are mainly found in the ovaries and surrounding the nucleus of the developing oocytes of female ticks (30 – 32). The *R. buchneri* genome encodes synthesis pathways to produce essential nutrients (biotin and folate) as well as for the production of anti-bacterial that could act to defend its tick host against invading bacteria (27). In fact, *R. buchneri* was shown to block tick cell superinfection with rickettsial pathogens *in vitro* (33), supporting this notion. However, their significance and potential impact on the fitness of *I. scapularis* are still largely unknown (34, 35). In this study, we investigated the interactions between the tick’s autophagy process and *R. buchneri*. We found that the expression profile of most autophagy family member proteins was down-regulated after *R. buchneri* infection. Autophagy was observed by autophagosome/autolysosome accumulation *in vitro* (tick cell culture) and *in vivo* (female tick ovary) in the presence of *R. buchneri*, while apoptosis was not induced. Inhibiting autophagy by siRNA interference could hamper intracellular rickettsial replication. This research on how *R. buchneri* affects its *I. scapularis* tick host will provide more clues to solve the tick-symbiont interaction puzzle and advance knowledge of tick biology.

## RESULTS

### Autophagy activation *in vitro* after *R. buchneri* infection

**Fig 1:**
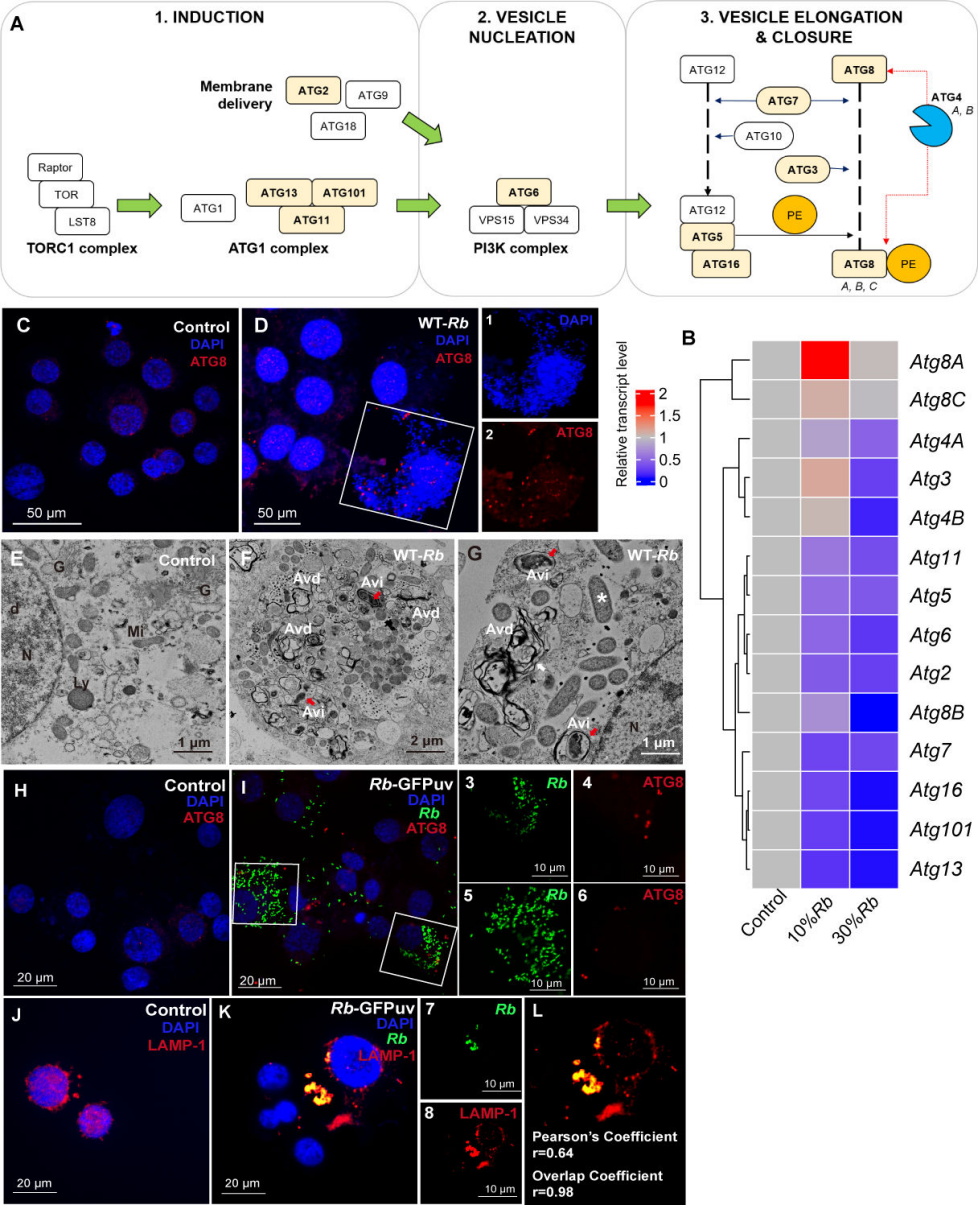
Autophagy activation *in vitro* after *R. buchneri* infection. (**A**) ATG in *I. scapularis* (highlighted in yellow), compared to *D. melanogaster*. (**B**) Expression profiles of *IsAtg* genes in IRE11 cells and *R. buchneri*-infected IRE11 cells (10% and 30% infection levels). The left clustering showed the gene expression patterns, and the heatmap represents different treatments (each column) and *IsAtg* expression relative to *gapdh* (each row). The color bars show the scale of relative expression levels, from red (high) to blue (low). (**C and D**) IRE11 and *R. buchneri*-infected IRE11 (10% infection) cells were fixed and labeled with anti-ATG8 antibody and secondary antibody conjugated to Alexa Fluor 594 (red). Blue indicates DAPI staining of the nuclei. *R. buchneri* infection induces autophagosome formation. The white box corresponds to enlarged panels, showing (1) Infected cells, DAPI stained nuclei, and *R. buchneri* individuals; ATG8 labeling the autophagosomes (2). Ultrastructure of IRE11 (**E**) and (**F and G**) *R. buchneri*-infected IRE11 (10% infection). Cells infected with *R. buchneri* displayed AVi with double membranes and AVd with multiple membrane-enclosed structures. The red arrows show degraded rickettsiae surrounded by double-membrane-bound AVi. The white arrows indicate multiple membrane-enclosed AVd. Golgi (**G**), Lysosome (Ly), Mitochondrion (Mi), and Nucleus (N). (**H and I**) Co-localization assay of *R. buchneri* and autophagosomes. IRE11 (**H**) and GFPuv transformed *R. buchneri*-infected IRE11 (10% infection) cells (**I**) were fixed and labeled with anti-ATG8 antibody and secondary antibody conjugated to Alexa Fluor 594 (red). Blue DAPI staining corresponds to the nuclei. The white boxes correspond to enlarged panels, showing *R. buchneri* (3, 5) and ATG8 labeling the autophagosomes (4, 6) in infected cells. (**J–L**) Co-localization assay of *R. buchneri* and lysosomes. IRE11 (**J**) and GFPuv transformed *R. buchneri*-infected IRE11 (10% infection) cells (**K**) were fixed and labeled with anti-LAMP-1 antibody and secondary antibody conjugated to Dylight 549 (red). Blue indicates DAPI staining of the nuclei; *R. buchneri* and LAMP-1-labelled lysosomes are shown separately in panels 7 and 8, respectively. (**L**) Pearson’s correlation coefficient (PCC) and overlap coefficient, according to MOC increased in co-localization of rickettsiae and lysosomes.

Our previous study identified 14 ATG in *I. scapularis* and showed these family members are highly conserved in ticks. An analysis of protein motif compositions indicated that the ATGs were evolutionarily closely related to their homologs in *D. melanogaster* where they are central to this pathway (Fig. 1A) (17). For instance, the ATG1 complex is recognized as the most upstream factor early in autophagy after starvation (36). We also found autophagy was activated after amino acid starvation in tick cells, confirming that the ATG1 complex has critical roles in initiating tick autophagy (17). To determine whether *R. buchneri* infection can trigger autophagy *in vitro*, we used a cell line from the European sheep tick, *Ixodes ricinus*, which has been shown to be superior to other cell lines for the growth and maintenance of *R. buchneri* (31). First, we examined the expression profiles of ATG genes in *R. buchneri*-infected IRE11 cells compared to uninfected controls. The proportion of *R. buchneri*-infected cells in culture was determined as previously reported (33). Considering the slow growth rate of *R. buchneri* (10–14 days population doubling time) in comparison to pathogens such as *Salmonella typhimurium* that triggered autophagy by damaging membranes at the early infection phase (hours), we used cultures in which 10% and 30% of the cells, respectively, were infected for this research (31, 37). Real-time PCR results showed a significant increase in *Atg8A* and *Atg8C* gene expression in cultures with 10% *R*. *buchneri* infected cells, whereas the expression of other *IsAtg* genes was reduced relative to uninfected control cells. In contrast, the expression of all *IsAtg* genes except *Atg8A* and *Atg8C* decreased significantly in the more heavily infected cultures (Fig. 1B; Fig. S1). Identification of autophagosomes by electron microscopy (EM) relies on the detection of the characteristic spherical structure enclosed by a double-layered membrane, and ATG8-family proteins facilitate membrane elongation during autophagosome formation (38). To verify that autophagy was induced by *R. buchneri* infection, we monitored the localization of ATG8-family proteins by immunofluorescence assays. Relative to uninfected IRE11 cells, *R. buchneri*-infected cells exhibited a remarkably increased localization of ATG8 to punctate structures representing autophagosomes (Fig. 1C and D). EM images also indicated a significant accumulation of autophagosomes, including initial autophagic vacuoles (AVi) with double-layered membrane and late/degradative autophagic vacuoles (AVd) with multiple membrane-enclosed structures (white arrows) relative to controls. We also found degraded *R. buchneri* (red arrow) surrounded by a double-layered membrane and cytoplasmic components (fragments of endoplasmic reticulum and Golgi) in AVi and AVd (Fig. 1E through G). This observation was further supported by the visualized autophagosomes, which were consistently present in most other cell samples (Fig. S3), confirming that autophagy was activated by *R. buchneri* infection. In addition, we found the presence of *R. buchneri* associated with autophagosomes by employing ATG8 (in red) and *R. buchneri* expressing GFPuv from a shuttle plasmid (*R. buchneri*-GFPuv; in green) (Fig. 1H and I). A similar overall effect was shown in *R. buchneri*-infected ISE6 cells (Fig. S2), i.e., *Atg8A* gene expression was significantly increased and more autophagosomes were observed compared to uninfected ISE6 cells. Thus, these data strongly suggest that *R. buchneri* infection (10%) triggered autophagosome formation in IRE11 cells. The later stage of autophagy involves the maturation of autophagosomes, which requires lysosome fusion, and this is where autophagosomes degrade and recycle cellular contents to supply nutrients (39). To further verify that autophagy was induced by *R. buchneri* infection, we examined lysosomal associated membrane protein 1 (LAMP-1, a lysosome marker) distribution after *R. buchneri-*GFPuv infection (Fig. 1J through L; Fig. S4). The degree of co-localization of *R. buchneri*-GFPuv (in green) and lysosome (in red) was analyzed by calculating Manders’ overlap Coefficient (MOC) (40), indicating the degree of co-localization of both fluorescence signals was positively correlated. Together, these results confirmed that autophagy induction is strongly related to *R. buchneri* infection *in vitro*.

### Altered plasma membrane structure in *R. buchneri* infected cells

**Fig 2:**
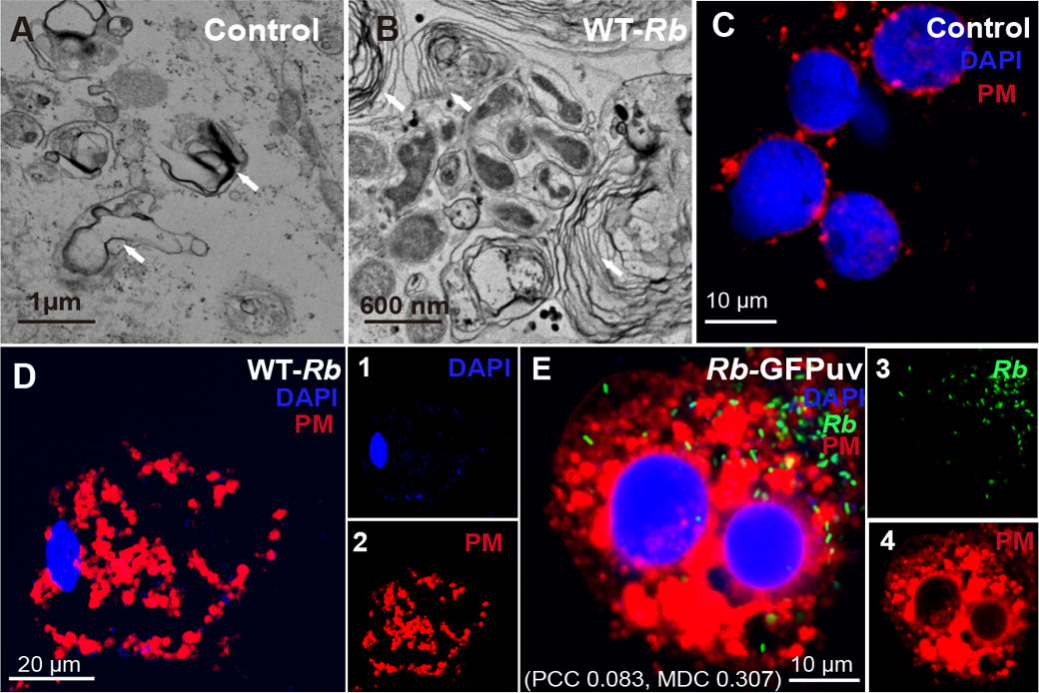
*R. buchneri* infection alters plasma membrane structure. Ultrastructure of (**A**) IRE11 and (**B**) *R. buchneri*-infected IRE11 (10% infection). Cells infected with *R. buchneri* displayed increased plasma membrane accumulation (indicated by white arrow). Live IRE11 cells (**C**), *R. buchneri*-infected IRE11 (**D**), and GFPuv transformed *R. buchneri*-infected IRE11 (**E**) were stained with Hoechst 33342 (blue) and CellMask Deep Red Plasma membrane dye (red). Panels 1–4 show infected cells: (1) Hoechst 33342-stained nuclei and *R. buchneri* individuals; *R. buchneri* (3), and plasma membrane (2, 4). PCC and overlap coefficient, according to MOC increased in co-localization of rickettsiae and plasma membrane.

Although multiple-layer membrane structures are a common feature of IRE11 cells (white arrows, Fig. 2A), *R. buchneri* infection induced much greater layer accumulation of these membrane structures, as seen using EM (Fig. 2B). To verify that *R. buchneri* infection induced this greatly amplified membrane accumulation, and to investigate the potential source of the membrane, we monitored the appearance of the plasma membrane in live cells by fluorescence microscopy. Compared with uninfected cells, *R. buchneri*-infected IRE11 (10% infection) displayed a greatly increased accumulation of plasma membrane, indicated by CellMask Deep Red Plasma membrane dye (red; Fig. 2C and D; Fig. S5). Additionally, *R. buchneri-*GFPuv (green) infected cells confirmed that accumulation of plasma membrane was associated with the presence of *R. buchneri* (Fig. 2E). Thus, these data strongly suggest that *R. buchneri* infection of IRE11 cells boosts the accumulation of multiple-membrane structures, which appear to be derived from the plasma membrane.

### No apoptosis induction after *R. buchneri* infection

**Fig 3:**
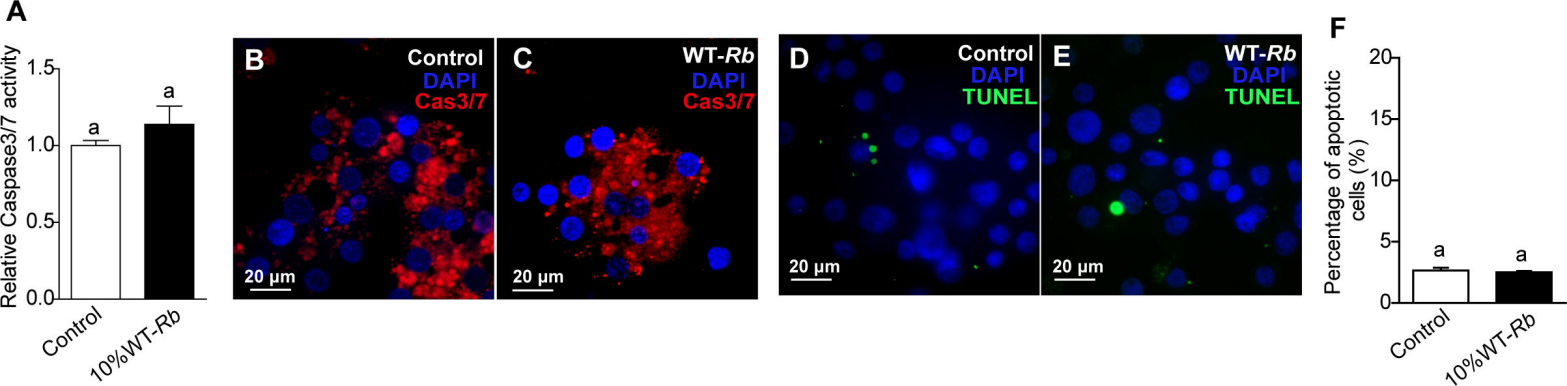
No apoptosis induction after *R. buchneri* infection. (**A**) Caspase3/7 enzyme activity in IRE11 and *R. buchneri*-infected IRE11 (10% infection) detected by the fluorescence plate reader. (**B and C**) *R. buchneri*-infected IRE11 (10% infection) cells showed no difference in red intensity representing caspase activity, relative to uninfected IRE11 cells. Blue DAPI staining corresponds to the nuclei. (**D and E**) IRE11 cells and *R. buchneri*-infected IRE11 cells (10% infection) were fixed and labeled with TUNEL (green). Blue DAPI staining corresponds to the nuclei. (**F**) Percentage of apoptotic cells (number of TUNEL-positive cells/number of DAPI-positive cells) in different treatments. In panels A and F, data are mean ± SD, and different letters above the columns indicate significant differences, *P* = 0.9496 (panel A), *P* = 0.1813 (panel B) (Student’s two-tailed *t*-tests).

Martins and colleagues demonstrated that *Rickettsia rickettsii* inhibited apoptosis in tick cells (41). In contrast, our previous study showed that the cleavage of caspase-3 promoted DNA fragmentation and led to apoptosis in *Rickettsia parkeri*-infected tick cells, suggesting species-specific interactions of rickettsiae with tick cells. With that research, we confirmed that Caspase3/7 activity can be measured with Magic Red dye, and that terminal deoxynucleotidyl transferase-mediated dUTP nick-end labeling (TUNEL) accurately identified DNA fragmentation in tick cells (42). When we measured the intensity of red fluorescence representing caspase activity (Fig. 3A; excitation wavelength of 592 nm), the plate reader showed no difference between control IRE11 and *R. buchneri*-infected IRE11 cells (10% infection level). Likewise, live cells stained with Magic Red dye showed no difference in red intensity representing caspase activity (Fig. 3B and C). To investigate whether DNA fragmentation was induced by *R. buchneri* infection, cells with different treatments were fixed and labeled with TUNEL, and images showed no difference in the percentage of apoptotic cells between uninfected and infected cells (Fig. 3D through F). These results confirm that there was no apoptosis induction after *R. buchneri* infection.

### siRNA cocktail targeting *Atg8A* and *Atg8C* inhibits *R. buchneri* replication

**Fig 4:**
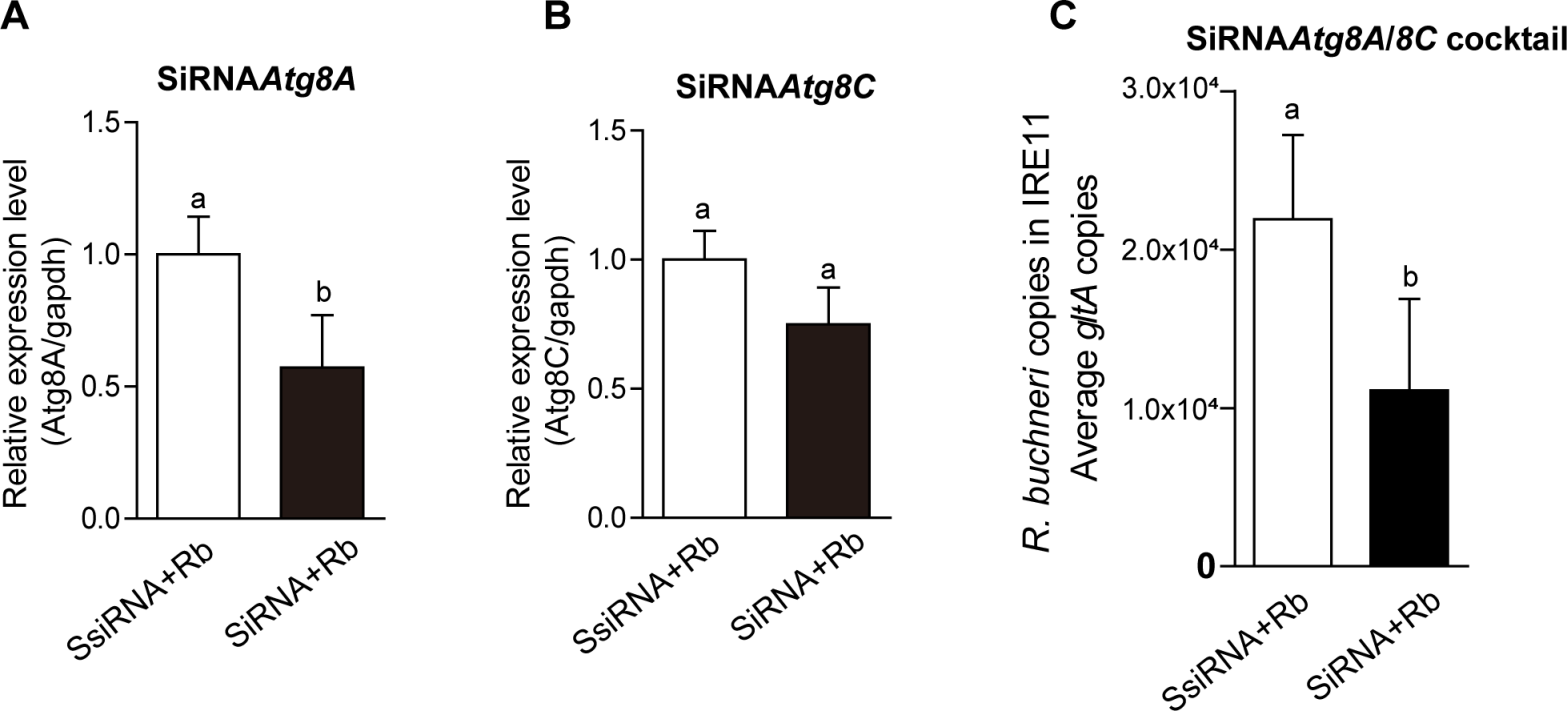
siRNA cocktail targeting *Atg8A* and *Atg8C* inhibits *R. buchneri* replication. *R. buchneri*-infected IRE11 (10% infection) cells were transfected with 160 nM *Atg8A*-silencing siRNA or 160 nM *Atg8C*-silencing siRNA and harvested for mRNA isolation 6 days after initiation of transfection, and 160 nM ssiRNA as control. Expression levels were normalized to *gapdh*. Relative expression of *Atg8A* (**A**) *and Atg8C* (**B**)*. R. buchneri*-infected IRE11 (10% infection) cells were transfected with the cocktail of 160 nM *Atg8A*-silencing siRNA and 160 nM *Atg8C*-silencing siRNA and harvested for DNA isolation 6 days after initiation of transfection. (**C**) *R. buchneri* genomic DNA abundance in IRE11 cells measured by quantitative PCR (qPCR) after siRNA treatments using *gltA* as a reference. In all panels, data are mean ± SD and different letters above the columns indicate significant differences, *P* = 0.0065 (panel A), *P* = 0.2780 (panel B), *P* = 0.0133 (panel C) (Student’s two-tailed *t*-tests).

Gene silencing in tick cell lines using siRNA to investigate the function of tick genes has been successful in some cell lines, including IRE11 (43). Given that ATG8 proteins are key players in regulating autophagosome formation and fusion with lysosomes, we target two *I. scapularis* ATG8 proteins via the cocktail of *Atg8A*-specific and *Atg8C*-specific siRNA, while the cocktail of *Atg8A*-scrambled and *Atg8C*-scrambled siRNA (ssiRNA) served as control. As shown in Fig. 4, significant knockdown of *Atg8A* gene expression in *R. buchneri*-infected IRE11 was achieved after 6 days of incubation with siRNA relative to ssiRNA, while the siRNA8C silencing experiment resulted in a slight reduction in gene expression without statistical significance. However, the cocktail of siRNAs resulted in an effective reduction of *R. buchneri* genomic DNA abundance (Fig. 4C). These results indicated that knockdown of the *Atg8A and Atg8C* gene inhibited *R. buchneri* intracellular replication in tick cells.

### *R. buchneri* association with autophagosomes and autolysosomes *in vivo*

**Fig 5:**
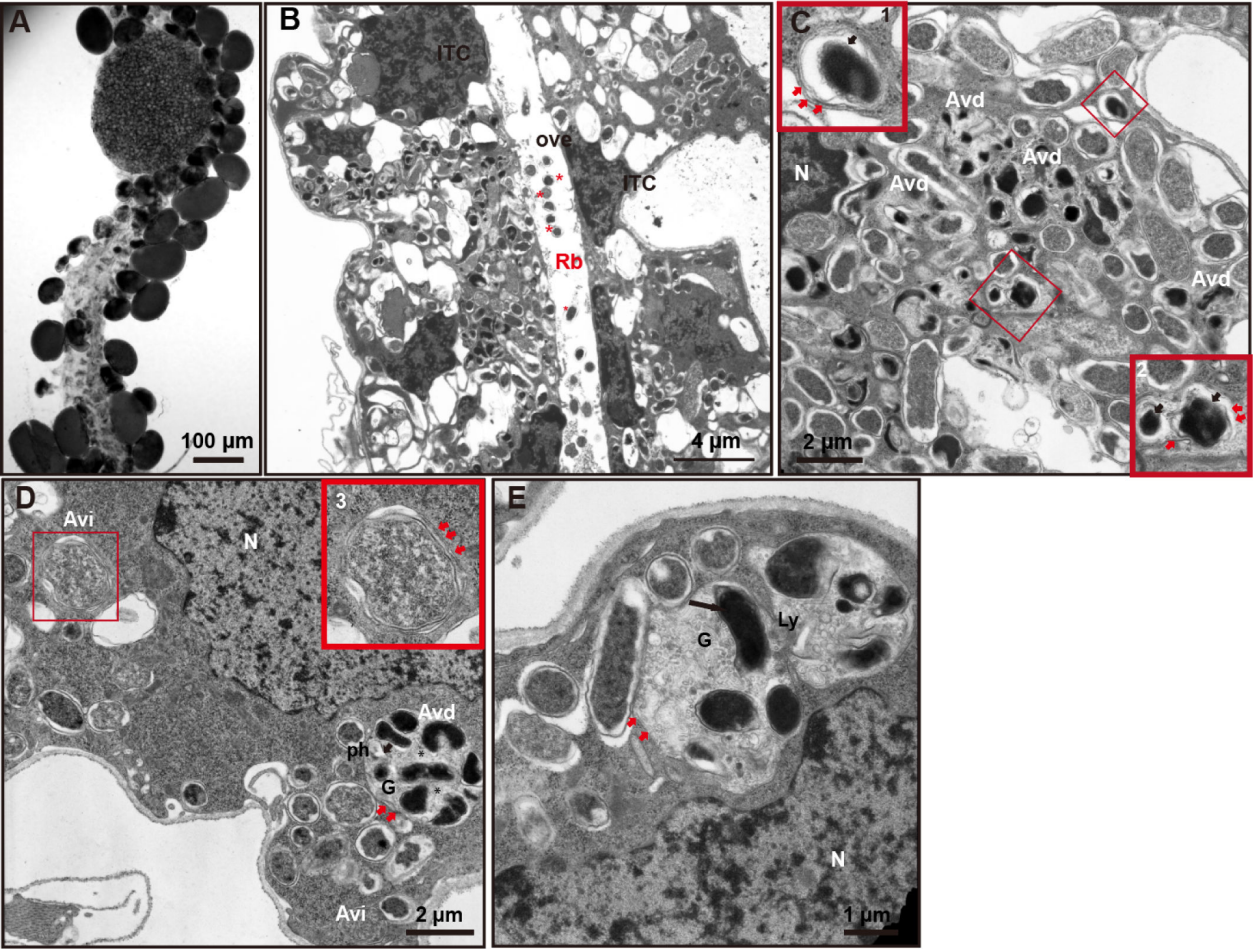
*R. buchneri* induce autophagosome formation *in vivo.* (**A**) Confocal micrograph of unfed female ovary under bright field microscopy. (**B**) Representative TEM images showing *R. buchneri* in tick ovaries. (**C**) TEM of *R. buchneri* in an interstitial cell: (1) The AVi/autophagosome containing *R. buchneri* (black arrow) with a double membrane (red arrows); (2) AVd/autolysosome containing degraded *R. buchneri* (black arrows) with a multi-membrane structure (red arrows). (**D**) Ultrastructure of oocytes. Phagophore (ph) containing fragments of cytoplasmic organelles, Golgi apparatus (G), and degraded *R. buchneri* were observed in the AVd (lower right). (3) The AVi/autophagosome. (**E**) An autophagosome with Golgi apparatus and *R. buchneri* merged with a lysosome in a developing oocyte.

We previously showed that *R. buchneri* resided in the ovaries and surrounded the nucleus of the developing oocytes of female ticks (31). Therefore, to determine whether *R. buchneri* infection can trigger autophagy *in vivo*, we used unfed female tick ovaries to observe autophagosome formation and fusion with lysosome by EM. As shown in Fig. 5A, partially developed ovaries displayed oocytes with prominent nuclei. Due to yolk granule accumulation, oocytes appeared dark when viewed using a confocal microscope and bright field illumination. EM images showed that *R. buchneri* mainly accumulated in the developing oocytes (Fig. S6A and B), and individual bacteria also disseminated to the ovary duct (indicated by *; Fig. 5B). Under high magnification, autophagosomes containing *R. buchneri* (black arrows) surrounded by double membranes (red arrows) (Fig. 5C, panel 1, Fig. S6C and D), and AVd/autolysosomes containing degraded *R. buchneri* (black arrows) with a multi-membrane structure (red arrows) were observed around the nucleus of an interstitial cell (Fig. 5C, panel 2). We also observed a typical autophagosome with the phagophore containing fragments of cytoplasmic organelles, Golgi apparatus, and degraded *R. buchneri* (Fig. 5D). In addition, an autophagosome containing Golgi apparatus and *R. buchneri* merged with a lysosome was seen in a developing oocyte (Fig. 5E). Autophagosomes were frequently observed in cells/tissues occupied by *R. buchneri*. These images provide evidence that *R. buchneri* may induce autophagosome and autolysosome formation *in vivo,* suggesting *R. buchneri* triggers autophagy but not xenophagy (a selective autophagy that specifically targets intracellular microbes to lysosomes).

## DISCUSSION

Although the significance of autophagy for the interactions of pathogens and their vectors is generally acknowledged, little is yet known regarding non-pathogenic microbes, let alone whether and how a vector’s autophagic pathway determines its fate. In this study, we show that infection of *I. scapularis* tick cell cultures by a symbiotic rickettsia induces autophagy and that the inhibition of autophagy impedes rickettsial intracellular growth, and this pathway is likely independent of apoptosis. We further identified two types of autophagy-related organelles (autophagosome/autolysosome) prevalent in female tick ovaries occupied by this rickettsia.

Autophagy-related genes and proteins are central to the autophagic process, and have been well-documented in eukaryotes, and we have also thoroughly characterized them in *I. scapularis* ticks (17). Although not all homologs encoding core ATG proteins in *D. melanogaster* have been found in *I. scapularis*, certain ATG proteins that contribute to autophagosome biogenesis were identified (Fig. 1A). For example, the initial step for autophagosome formation is the nucleation of the phagophore, which is regulated via the ULK1/2 complex (44), and ULK1/2 (or ATG1), ATG13, FIP200 (or ATG11), and ATG101 are recognized as critical components for this complex in mammals. It is not surprising that ticks lack ULK1/2 but can still induce autophagosome formation (Fig. 1) because some ATG proteins can compensate for or replace others in the recruitment of ATG components (45, 46). Additionally, EM showed autophagosome (with *R. buchneri* inside) fusion and cargo degradation, suggesting this represented the later phase of autophagy in 10% *R*. *buchneri*-infected cultures. An examination of the expression profile of 14 *Atg* genes showed that transcripts from eight of them were significantly reduced, but not for two *Atg8* subfamily members (8A and 8C) (Fig. 1), as well as *Atg3*. However, higher infection (30%) with *R. buchneri* decreased the transcription of most *Atg* genes (Fig.S1 and S2). We suspect that (i) *R. buchneri* induced the later phase of autophagy and decreased *A*tg gene transcription, thereby balancing the tick autophagic process for the benefit of the bacteria, and (ii) different infection levels led to different outcomes of the autophagy response. This hypothesis is supported by the finding that *Listeria monocytogenes* activate autophagy at the early stage of infection in order to evade containment in autophagosomes at the later infection stage (47, 48). Similarly, *R. parkeri* leads to apoptosis inhibition at early stages of infection but activates apoptosis during late infection (42). Unlike the well-known systems of pathogenic bacteria and their hosts, examining tick *in vivo* autophagy in response to *R. buchneri* is very challenging because of strict intracellular growth requirements and slower growth rates compared to other *Rickettsia* spp. (49). Although *R. buchneri* infection level could be a factor that initiates different responses *in vitro* (33, this manuscript), future studies using infection by host-cell free *R. buchneri* instead of infected cells (49), may help to determine the specific requirements (such as a multiplicity of infection and cell types) that regulate tick autophagy at different phases.

Finding the balance between avoidance of autophagy and successful replication is necessary for the survival of intracellular bacteria, given that autophagy is an innate immune response to target bacteria for degradation and that bacteria are evolving strategies to subvert autophagy for their own needs (50). Although much progress has been achieved in elucidating the mammalian host autophagy response to certain tick-borne intracellular bacterial pathogens, such as *A. phagocytophilum*, *Ehrlichia chaffeensis,* and *R. parkeri,* this is still completely unknown in their tick vectors (51 – 53). For example, *A. phagocytophilum* used the effector protein Ats-1 to employ autophagosomes to promote their intracellular growth and block the fusion of the autophagosome with the lysosome. By contrast, *R. buchneri* infection led to autophagosome formation and fusion with lysosomes. The co-localization of *R. buchneri* and lysosomal marker also indicated a different mechanism by which non-pathogenic bacteria interfere with tick autophagy (Fig. 1). This is also confirmed by spotted fever group (SFG) rickettsia behavior in human macrophage-like cells, in which symbiotic or non-pathogenic rickettsiae colocalize with lysosomal markers, whereas pathogenic rickettsiae evade lysosomal destruction (54, 55). This suggests that endosymbiotic *Rickettsia* species are cleared by autophagy as part of the mammalian immune response. It is unclear whether the induction of late-stage autophagy and lysosomal degradation of *R. buchneri* in ticks is an immune response to control the symbiont to numbers that are tolerable to the tick, or whether autophagy is induced by *R. buchneri* to aid tick survival, for example, as a mechanism to obtain nutrients. *R. buchneri*-induced autophagy appeared to be a non-selective process, with *R. buchneri-*containing autophagosomes also engulfing cellular cytoplasmic contents for lysosomal degradation, including Golgi and mitochondria (Fig. 5). This differs from xenophagy, a selective form of autophagy which specifically targets intracellular pathogens to lysosomes and mediates their destruction, and would be expected to occur if *R. buchneri-*induced autophagy were a targeted immune response. It is possible that in heavily infected cells that are packed with *R. buchneri* (as can be seen in Fig. 1 and 5), activating non-selective autophagy in tick cells leads to the unavoidable engulfment and destruction of some *R. buchneri* whilst the remaining multitude of bacteria receives the benefits accompanying increased autophagy. It should be noted that most of the results presented here were obtained in tick cells, which could have different immune responses compared to the symbiont’s usual home in the tick ovaries. However, our findings suggest that *R. buchneri* infection in both tick cells and ovaries triggered similar responses, in terms of inducing autophagosome formation. Therefore, autophagy may serve as a strategic option for *R. buchneri*, whether it’s activated or not, depending on the advantages it provides to the bacteria, especially during different phases such as during or after a bloodmeal. We are uncertain of how *R. buchneri* induces autophagy and whether this involves interactions between bacterial factors (such as effectors) and tick ATG proteins. Some *R. buchneri* proteins are recognized as potential effectors that may be secreted via the type IV secretion system (34). We used RNA interference (RNAi) as a powerful tool to demonstrate that ATG8 protein expressed during autophagy promotes rickettsial intracellular replication (Fig. 4). Although the reduction in *R. buchneri* replication upon the cocktail of *Atg8A*/8C siRNA treatment was relatively small, it was significant, and the effects on *R. buchneri* growth over time would be even greater considering its slow doubling rate of approximately 10 days (31, 33). It should be noted that the siRNA8C silencing experiment resulted in a slight reduction in gene expression without statistical significance, we speculated that ATG8C protein may have a different expression pattern after *R. buchneri* infection. Furthermore, the functional redundancy of the three *I. scapularis* ATG8 proteins and whether they act sequentially or in concert during autophagosome formation are unknown; thus, it is possible that upon knockdown of *Atg8A,* the other two ATG8 proteins may have compensated for the loss of *Atg8A* activity, resulting in a smaller effect on *R. buchneri* replication than if all *Atg8* genes had been targeted. This is common in mammals where the ATG8 family comprises six members, including microtubule-associated protein 1 light chain 3 (LC3) and GABARAP subfamilies. LC3 isoforms partially compensate for LC3 loss, and GABARAP isoforms are recognized as redundant proteins that restore autophagy *in vitro* (56). Further study to define the function of tick ATG proteins will be necessary to identify the basic autophagy machinery in ticks. This will provide the basis for exploring *R. buchneri* effectors that may interfere with tick autophagy and determine whether they function in an SFG rickettsia-specific manner or trigger generally conserved responses in their tick vectors.

We also observed some unique features during *R. buchneri*-induced autophagy processes, such as accumulation of plasma membrane (indicated by white arrow) and an increased appearance of multi-membrane structures (Fig. 2), compared to uninfected tick cells (shown here in IRE11 cells). This multiple-membrane structure might be a potential source for the autophagosomal membrane in ticks (57), as plasma membrane, as well as the endoplasmic reticulum, the Golgi, and the mitochondria, can supply membrane lipids for autophagosome formation; however, the relationship between the plasma membrane and this structure needs further investigation.

Considering that autophagy and apoptosis are integrated and have been implicated in microbial infections, we monitored caspase activity and DNA fragmentation and found no notable variation, suggesting *R. buchneri* employs distinct strategies to activate tick autophagy and apoptosis (Fig. 3). This is probably because endosymbionts should preferentially induce the cell survival pathway (autophagy) to benefit their intracellular growth, instead of the death pathway (apoptosis), which would be detrimental to them. Our lab previously demonstrated that *R. parkeri*, a pathogenic SFG rickettsia, induced tick apoptosis *in vitro* (42). Apparently, the species-specific behavior of rickettsiae can drive different outcomes of apoptosis, even though both *R. buchneri* and *R. parkeri* belong to the SFG. Additionally, it is not known whether there is cross-talk between autophagy and apoptosis. This cross-talk could govern the cells' fate in response to pathogen stimuli, thus affecting pathogen destiny, i.e., their ability to successfully complete their life cycle through infection, replication, and transmission. For example, *Coxiella burnetii* can generate a persistent bacterial infection by preventing apoptosis and inducing autophagy through interaction with Bcl-2 and Beclin-1 (58, 59). The knowledge gained from the communication between autophagy and apoptosis will significantly enhance our understanding of the defense responses that regulate pathogen infection and burden in vector ticks. Here, we revealed that a symbiotic rickettsia induces autophagy *in vitro* and *in vivo* through morphological and genetic methods and demonstrated that autophagy restricted intracellular endosymbiont replication. This finding advances our knowledge of the biology of symbiotic rickettsiae and their impact on arthropod hosts, and sheds light on the basic autophagy machinery of ticks and other arthropod vectors.

## MATERIALS AND METHODS

### Cell cultures and *Rickettsia* growth

*I. scapularis* and *I. ricinus* tick cell lines (ISE6 and IRE11) were cultured in complete medium (L-15C300, 5% fetal bovine serum (FBS), 5% tryptose phosphate broth, and 0.1% lipoprotein concentrate) at 34°C (42). *R. buchneri* ISO7^T^ (referred to as WT-*R. buchneri*) (60) and transformed *R. buchneri* expressing GFPuv from the plasmid pRAM18dRGA (referred to as *R. buchneri*-GFPuv) (60) were cultured in IRE11 or ISE6 at 28°C in the above complete medium but with 10% FBS and additionally supplemented with NaHCO_3_ and HEPES buffer (33). The infection level of *R. buchneri* in tick cells was assessed via Giemsa staining as reported previously (33).

### RNA extraction and real-time quantitative reverse transcription PCR (qRT-PCR)

Total RNA was extracted from cells using TRI Reagent (Sigma, St. Louis, MO, USA), and purified by an RNA Clean & Concentrator kit (Zymo Research, Irvine, USA). The quantity and quality of RNA were assessed via a DS-11 Series Spectrophotometer/Fluorometer (DeNovix, Wilmington, USA). Genomic DNA contamination was eliminated and cDNA was synthesized using PrimeScript RT Reagent Kit with gDNA Eraser (Takara, Otsu, Shiga, Japan). The expression profile of tick ATG (17) at different infection rates was assessed using qPCR on the Mx3005P Real-Time system (Stratagene, La Jolla, USA) with SYBR green detection (Agilent Technologies, Santa Clara, USA). All protocols were according to the manufacturer’s instructions. Primers used in this study are listed in Table S1. The analysis of the *Atg* gene expression profile included a one-way analysis of variance for each gene, with subsequent Bonferroni correction to address multiple comparisons, and the glyceraldehyde 3-phosphate dehydrogenase (*gapdh*) gene from the *I. scapularis* tick genome as a reference control. Relative gene expression levels of individual *Atg* genes were calculated using the comparative cycle threshold (CT) method (2−^ΔΔ^CT). Statistical analysis was performed using the data obtained from three separate cDNA sets from three independent biological samples.

### *R. buchneri* genomic DNA isolation and qPCR

Total genomic DNA was isolated from WT-*R. buchneri* infected cells using the Qiagen Puregene Core A kit, following the manufacturer’s gram-negative bacteria instructions. The quantity and quality of genomic DNA were assessed via a DS-11 Series Spectrophotometer/Fluorometer. The single-copy rickettsial citrate synthase (*gltA*) gene was used to determine the copies of WT-*R. buchneri* in different treatments using qPCR as previously described (61). Primers used in this study are listed in Table S1.

### Immunofluorescence

WT-*R. buchneri* or *R. buchneri*-GFPuv infected cells (10% infection) and uninfected control cells were immobilized onto slides and fixed in 4% paraformaldehyde for 1 h at room temperature. They were then permeabilized with 0.1% Tween 20 (in 1× PBS) for 1 h, blocked in 5% BSA for 2 h and subsequently incubated with primary antibody overnight at 4°C.

The slides were then washed twice in TBST (10 mM Tris-HCl, 150 mM NaCl, 0.05% Tween-20, pH 7.5) and incubated with secondary antibody for 2 h at room temperature. They were then mounted in Fluoroshield mounting medium with DAPI (VECTASHIELD, Vector Laboratories), and imaged under a Nikon A1si Spectral Confocal Microscope using a 60X objective or an Olympus BX61 Disk Scanning Unit confocal microscope fitted with a 60X objective. The anti-LC3B antibody (purchased from Abcam, Cambridge, UK) was used as the primary antibody at 1:2,000 dilution for staining ATG8 protein in cells. The anti-LAMP1 antibody (purchased from Cell Signaling Technology, Danvers, MA, USA) was the primary antibody at 1:2,000 dilution for staining lysosome/autolysosome in cells. A goat anti-rabbit IgG (Alexa Fluor 594) was used as the secondary antibody at a 1:3,000 dilution (purchased from Abcam). The fluorescence properties were observed using different wavelength filters (4, 6-diamidino-2-phenylindole (DAPI): excitation at 365 nm and emission at 480 nm; FITC: excitation at 495 nm and emission at 519 nm; TRITC: excitation at 560 nm and emission at 590 nm; Cy5: excitation at 647 nm and emission at 665 nm) depending on the fluorophores used for secondary antibodies. All treatments were replicated three times. Images were processed using the program NIS-Elements View 4.50 (University Imaging Centers at the University of Minnesota, Twin Cities). The colocalization of *R. buchneri* and lysosomes was assessed by determining the degree of the area of two signals overlapping, according to Pearson’s coefficient and overlap coefficient according to Manders’ calculations by ImageJ Fiji (JaCoP plugin and Colocalization Threshold plugin) (62).

### Transmission electron microscopy

WT-*R. buchneri*-infected tick cells (10% infection in IRE11) and uninfected control cells were pre-fixed with 2.5% glutaraldehyde at 4°C over three nights. They were rinsed in 1× PBS three times for 10 min and post-fixed in 2% OsO_4_ for 2 h. Samples were rinsed in 1× PBS three times for 10 min and dehydrated in a graded ethanol series. For 25% and 50%, the samples were rinsed once for 10 min. For 75%, 95%, and 100%, the samples were rinsed twice for 10 min. Subsequently, the samples were rinsed twice in 100% acetone for 10 min and infiltrated in 50% Embed 812 resin/acetone solutions overnight. Then the solvent was replaced with 100% Embed 812 resin/BDMA (N-Benzyl-N, N-Dimethylamine C_6_H_5_CH_2_N(CH_3_)_2_) twice for 12 h at room temperature. Next, the samples were polymerized at 60°C for 48 h. Finally, the pieces were cut into semi-thin sections (toluidine blue stain for 2–5 min) and thin sections (uranyl acetate stain for 15 min and lead citrate stain for 5 min) and examined using a JEM-1400Plus TEM.

To examine tick ovaries, the dorsal cuticle was dissected away from three unfed *I. scapularis* female ticks (from a laboratory colony maintained at the University of Minnesota) and immersed in Ito’s modified fixative (63) for pre-fixation. They were then rinsed in 1× PBS three times for 10 min and post-fixed in 1% OsO_4_ in 0.4M sodium cacodylate buffer for 1 h. Secondary fixation used 2% uranyl acetate, en bloc for 1 h. First, the samples were rinsed in 1× PBS three times for 10 min and dehydrated in a graded ethanol series (25%, 50%, 75%, and 95%) for 10 min. Then the samples were rinsed twice in 100% ethanol for 10 min and infiltrated in 25%, 50%, and 75% Spurr’s low-viscosity resin/ethanol in each solution for 24 h. Then the solvent was replaced with 100% Spurr’s low viscosity resin for 24 h at room temperature. Next, the samples were polymerized at 70°C for 8–12 h. Finally, the pieces were cut into semi-thin sections (toluidine blue stain for 3–4 min) and thin sections (uranyl acetate stain for 20 min and modified Sato’s lead solution stain for 5 min) examined using Philips CM12 TEM.

### Plasma membrane staining

Tick cell plasma membranes were stained using the CellMask Plasma Membrane Stains Kit (Thermo Fisher Scientific), following the manufacturer’s instructions. WT-*R. buchneri* or *R. buchneri*-GFPuv infected cells (10% infection in IRE11) and uninfected control cells were incubated with Deep Red plasma membrane stain substrate and NucBlue Live ReadyProbes Reagent (Hoechst 33342) (Thermo Fisher Scientific), and then immobilized onto slides. Specimens were mounted in a Fluoroshield mounting medium and imaged under a Nikon A1si Spectral Confocal Microscope using a 60X objective. Dual fluorescence properties were observed using filters (DAPI and Cy5) as above. All treatments were replicated three times. Images were processed using the program NIS-Elements View 4.50 (University Imaging Centers at the University of Minnesota, Twin Cities). The mean of fluorescence intensity and a correlation analysis between the intensity of red and green staining were performed using ImageJ Fiji.

### Monitoring apoptosis in response to *R. buchneri* infection in tick cells

Caspase enzyme activity assay (Magic Red Caspase3/7 Assay Kit, Immunochemistry), and DNA fragmentation via TUNEL (*in situ* Cell Death Detection Kit, Roche) were applied to monitor apoptosis in WT-*R. buchneri*-infected cells (10% infection in IRE11), and uninfected cells served as controls, following the protocols previously reported (42). For each treatment, TUNEL-positive cells and DAPI-positive cells in a total of three slides were quantified. From each slide, three fields were randomly selected, and the percentage of apoptotic cells was calculated as the number of TUNEL-positive cells divided by the number of DAPI-positive cells in these chosen fields. The student’s two-tailed *t*-test was applied for the analysis of percentages of apoptotic cells. All treatments were replicated three times.

### RNA interference

The *I. scapularis Atg8A* and *Atg8C* sequence (gene ID: ISCW000710-RA, gene ID: ISCW017654-RA) are accessible at VectorBase (https://www.vectorbase.org/organisms/ixodes-scapularis). siRNA primers targeting *I. scapularis Atg8A* and *Atg8C* were designed by BLOCK-iT RNAi Designer (Thermo Fisher Scientific) and are listed in Table S1. Primers were searched against the *I. scapularis* genome database using BLASTN to evaluate target-specificity. Scrambled and *Atg8A* or *Atg8C-*specific siRNA were synthesized using the Silencer siRNA Construction Kit (Thermo Fisher Scientific), following the manufacturer’s protocols. Lipofectamine 3,000 Transfection Reagent was used for the transfection of siRNA into tick cells (Thermo Fisher Scientific). Uninfected and *R. buchneri* infected cells were transfected with 160 nM siRNA (or 160 nM ssiRNA as control) and harvested for mRNA isolation 6 days after initiation of transfection. The cocktail of *Atg8A*-specific and *Atg8C*-specific siRNA (ssiRNA as control) were transfected into *R. buchneri* infected cells and harvested for mRNA isolation 6 days after initiation of transfection. *Atg8A* and *Atg8C* gene relative expression were detected by qRT-PCR and normalized to *gapdh. R. buchneri* genomic DNA was measured by qPCR and normalized to citrate synthase-encoding gene (*gltA*) after siRNA treatments as previously described (42). The student’s two-tailed *t*-test was applied to analyze the quantities of *R*. *buchneri* DNA and the relative gene expression.

## SUPPLEMENTAL MATERIAL

Fig. S1 spectrum.01086-23-s0001.tif Relative expression of *IsAtgs* in IRE11 and *R. buchneri*-infected IRE11 (10% and 30% infection rates) cells, relative to *gapdh*.

Fig. S2 spectrum.01086-23-s0002.tif Autophagy activation in ISE6 cells after *R. buchneri* infection.

Fig. S3 spectrum.01086-23-s0003.png Ultrastructure of *R. buchneri*-infected IRE11 (10% infection).

Fig. S4 spectrum.01086-23-s0004.tif GFPuv transformed *R. buchneri*-infected IRE11 (10% infection) cells were fixed and labeled with anti-LAMP-1 antibody and secondary antibody conjugated to Dylight 549 (red).

Fig. S5 spectrum.01086-23-s0005.tif Fluorescence intensity assay.

Fig. S6 spectrum.01086-23-s0006.png Representative TEM images showing *R. buchneri* in tick ovaries.

Supplemental legends spectrum.01086-23-s0007.docx Legends for Fig. S1 to S6.

Table S1 spectrum.01086-23-s0008.docx Primers in this study.

ASM does not own the copyrights to Supplemental Material that may be linked to, or accessed through, an article. The authors have granted ASM a non-exclusive, world-wide license to publish the Supplemental Material files. Please contact the corresponding author directly for reuse.
