# Supplementary figures and images for: The role of autophagy in tick-endosymbiont interactions: insights from Ixodes scapularis and Rickettsia buchneri
Source: Microbiol Spectr. 2023 Dec 1;12(1):e01086-23. doi: 10.1128/spectrum.01086-23 (PMC10783069; doi:10.1128/spectrum.01086-23)

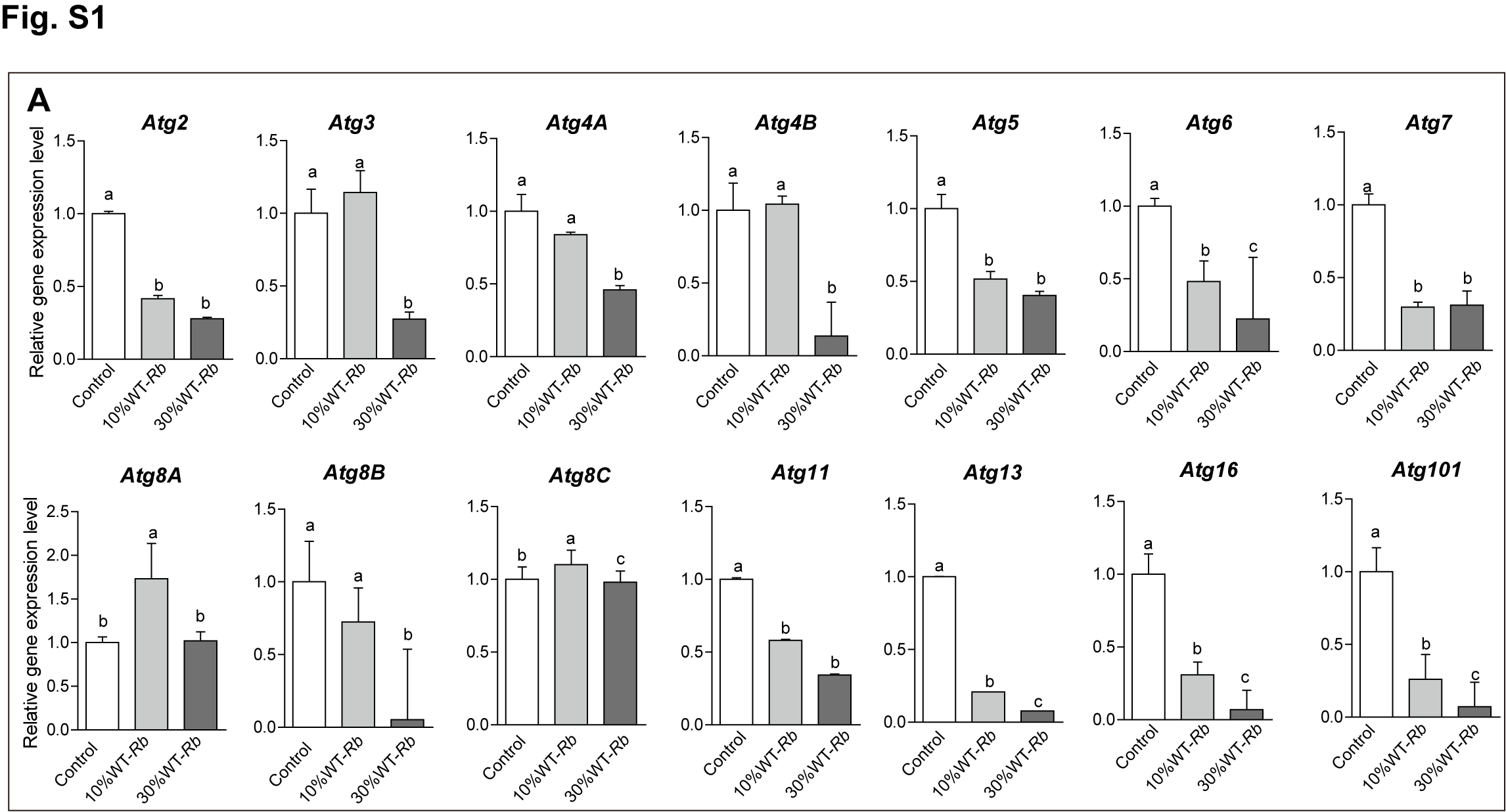

Supplement: Fig. S1 — Relative expression of IsAtgs in IRE11 and R. buchneri-infected IRE11 (10% and 30% infection rates) cells, relative to gapdh. [file spectrum.01086-23-s0001.tif]

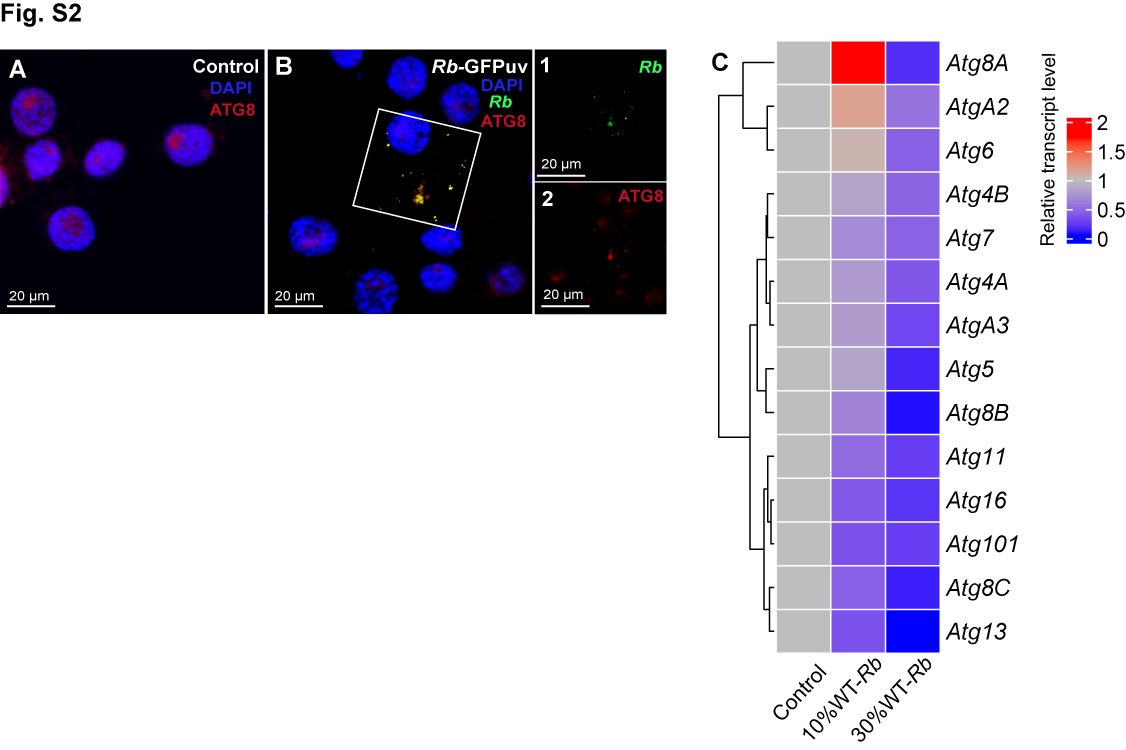

Supplement: Fig. S2 — Autophagy activation in ISE6 cells after R. buchneri infection. [file spectrum.01086-23-s0002.tif]

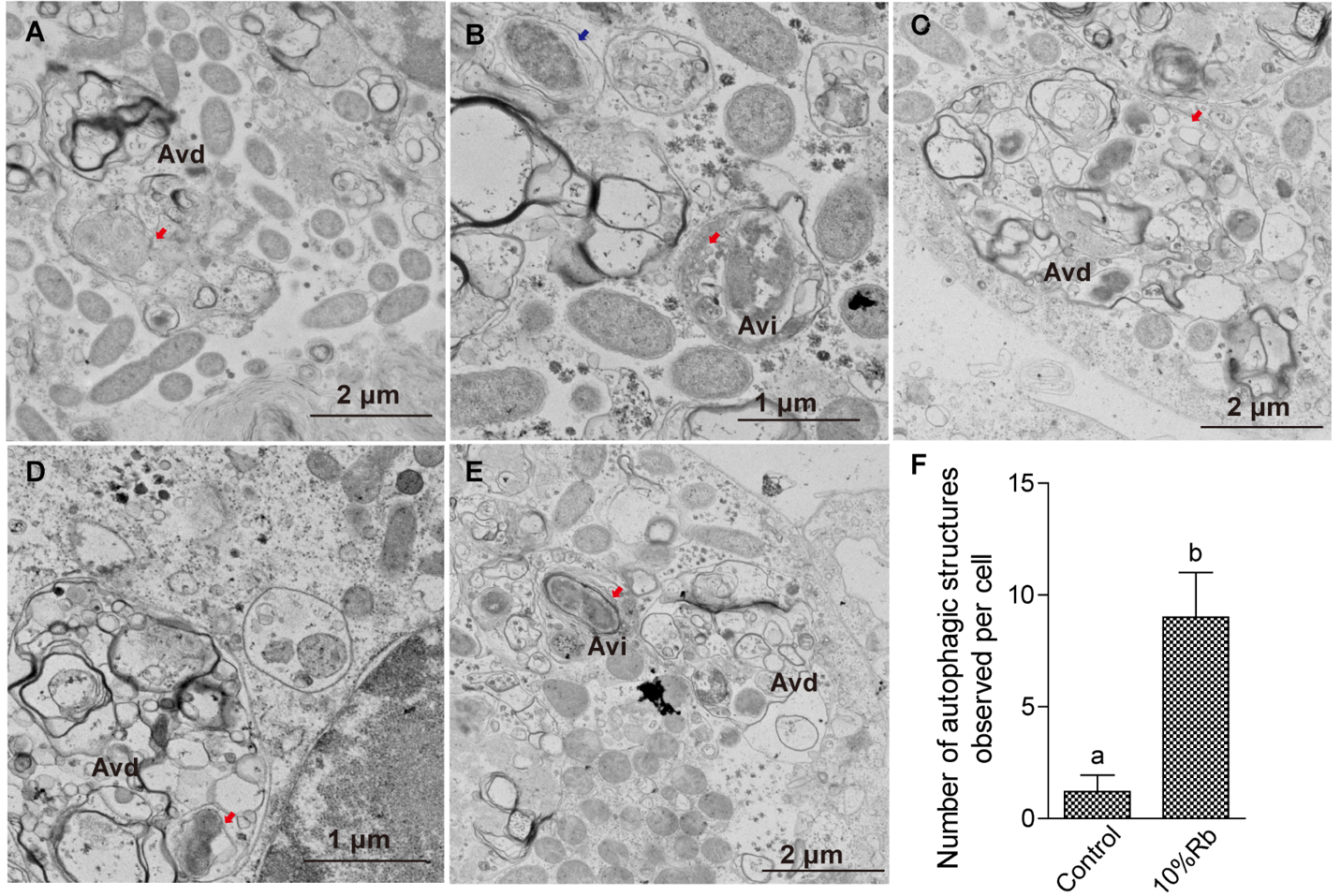

Supplement: Fig. S3 — Ultrastructure of R. buchneri-infected IRE11 (10% infection). [file spectrum.01086-23-s0003.png]

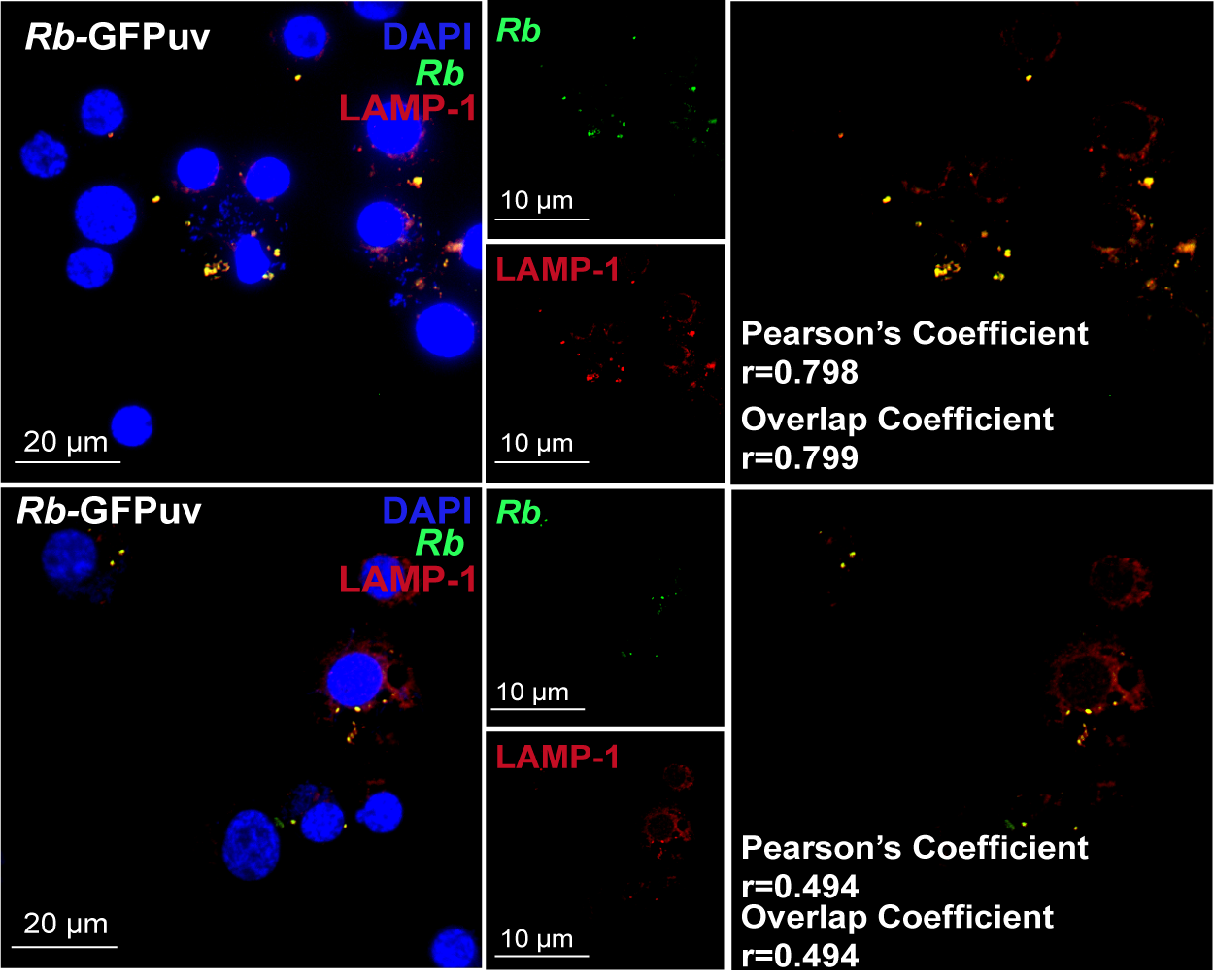

Supplement: Fig. S4 — GFPuv transformed R. buchneri-infected IRE11 (10% infection) cells were fixed and labeled with anti-LAMP-1 antibody and secondary antibody conjugated to Dylight 549 (red). [file spectrum.01086-23-s0004.tif]

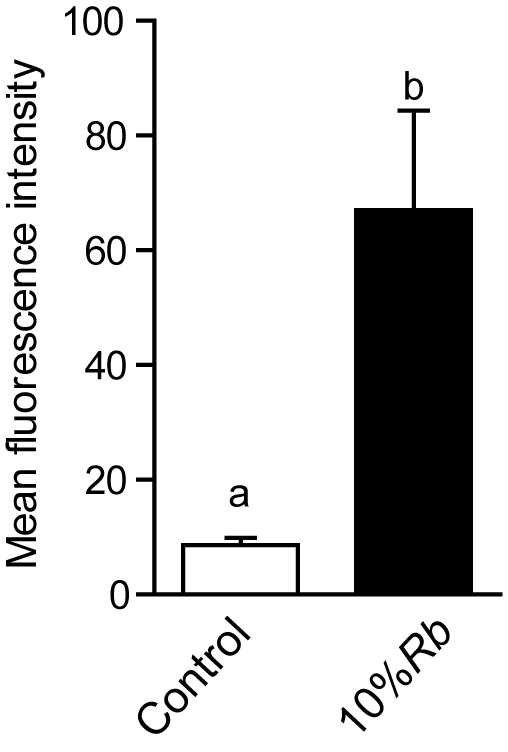

Supplement: Fig. S5 — Fluorescence intensity assay. [file spectrum.01086-23-s0005.tif]

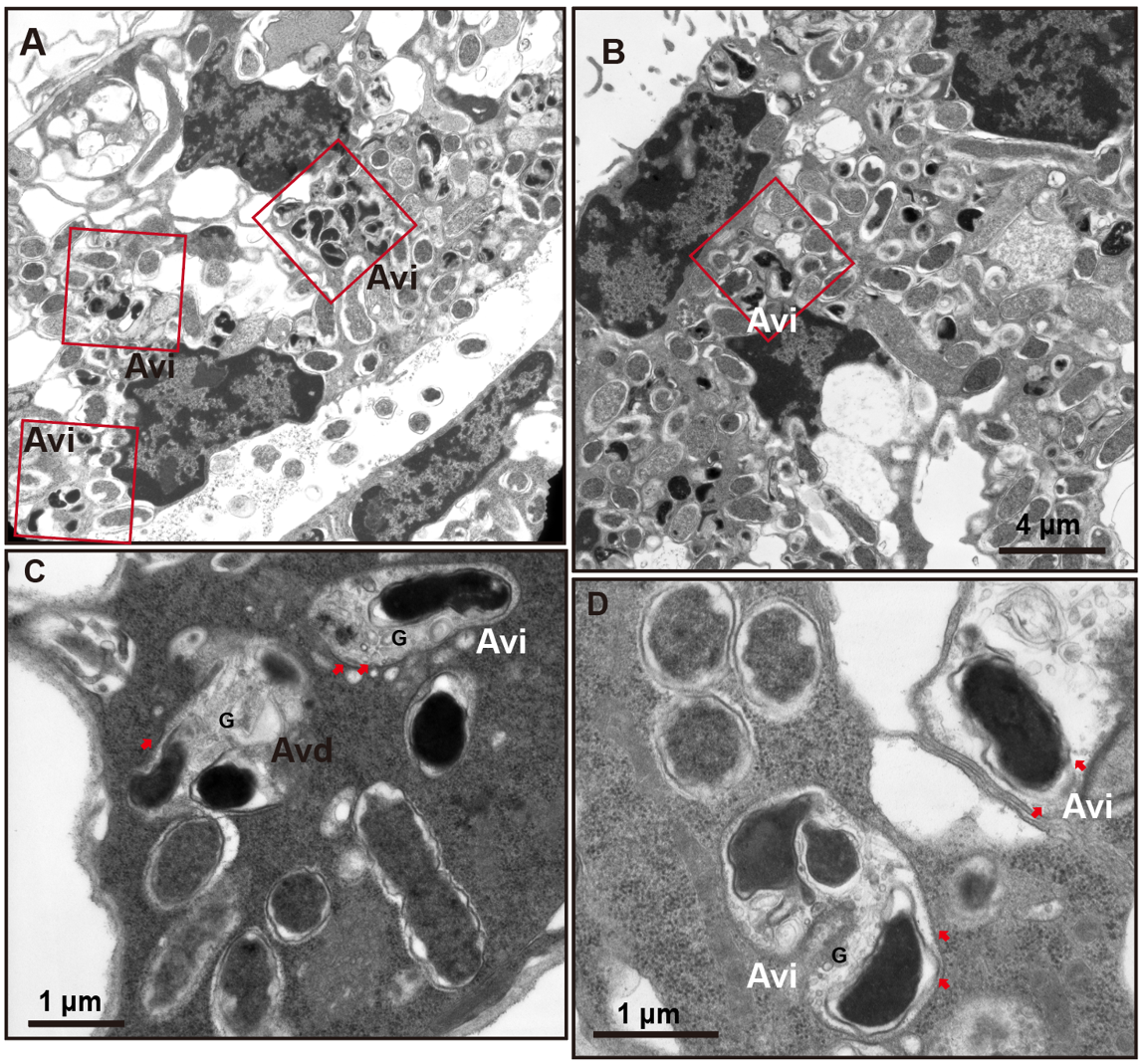

Supplement: Fig. S6 — Representative TEM images showing R. buchneri in tick ovaries. [file spectrum.01086-23-s0006.png]
