# Supplementary material for: The role of autophagy in tick-endosymbiont interactions: insights from Ixodes scapularis and Rickettsia buchneri
Source: Microbiol Spectr. 2023 Dec 1;12(1):e01086-23. doi: 10.1128/spectrum.01086-23 (PMC10783069; doi:10.1128/spectrum.01086-23)
Supplement: Supplemental legends — Legends for Fig. S1 to S6. [file spectrum.01086-23-s0007.docx]

**FIGURE LEGENDS**

**Fig.S1** Relative expression of *IsAtgs* in IRE11 and R. buchneri-infected IRE11 (10% and 30% infection rates) cells, relative to *gapdh*. Data are mean ± SD, and different letters above the columns indicate significant differences, p < 0.05 (ANOVA, followed by Bonferroni test).

**Fig.S2** Autophagy activation in ISE6 cells after *R. b*uchneri infection. ISE6 (**A**) and GFPuv transformed R. buchneri-infected ISE6 (10% infection) cells (**B**) were fixed and labeled with anti-ATG8 antibody and secondary antibody conjugated to Alexa Fluor® 594 (red). Blue DAPI staining corresponds to the nuclei. The white box indicates enlarged panels, showing R. buchneri (**1**) and ATG8-labelled autophagosomes (**2**) in infected cells. (**C**) Expression profiles of IsAtg genes in ISE6 cells and R. buchneri-infected ISE6 cells (10% and 30% infection levels). The left clustering shows the gene expression patterns, and the heatmap represents results from different treatments (each column) and IsAtg expression (each row). The color bars show the scale of relative expression levels, from red (high) to blue (low).

**Fig.S3** Ultrastructure of R. buchneri-infected IRE11 (10% infection). Cells infected with *R. buchneri* displayed initial autophagic vacuoles (AVi) with double membranes and degradative autophagic vacuoles (AVd) with multiple membrane-enclosed structures. The red arrow (**A**) shows degraded endoplasmic reticulum surrounded by double-membrane-bound AVd. (**B**) Phagophore containing Golgi apparatus (red arrow), and degraded *R. buchneri* (blue arrow) were observed in the AVi. (**C-D**): Cells infected with *R. buchneri* show the presence of AVd structures, a complex multiple-membrane structure within AVd is indicated by the red arrow (**C**), and a degraded mitochondrion within an AVd is shown in (**D**). (**E**) Cells infected with *R. buchneri* display both AVi and AVd structures, as highlighted by the red arrows. (**F**)

Numbers of autophagic structures observed per cell in both IRE11 and *R. buchneri*-infected IRE11 cells (10% infection) revealed a significant difference (p = 0.0008) in a total of five randomly selected cells. The data are presented as mean ± SD, and different letters above the columns indicate significant differences (p < 0.05, Student’s two-tailed t-tests).

**Fig.S4** GFPuv transformed R. buchneri-infected IRE11 (10% infection) cells were fixed and labeled with anti-LAMP-1 antibody and secondary antibody conjugated to Dylight 549 (red). Blue indicates DAPI staining of the nuclei; Pearson’s correlation coefficient (PCC) and Manders overlap coefficient (MOC) increased during co-localization of rickettsiae and lysosomes.

**Fig.S5** Fluorescence intensity assay. Fluorescence intensity assays conducted in both IRE11 and *R. buchneri*-infected IRE11 cells (10% infection) revealed a significant difference (p = 0.0443) in red intensity compared to uninfected IRE11 cells in a total of three randomly selected fields. The data are presented as mean ± SD, and different letters above the columns indicate significant differences (p < 0.05, Student’s two-tailed t-tests).

**Fig.S6** Representative TEM images showing *R. b*uchneri in tick ovaries. (**A-B**) Cells infected with *R. buchneri* display initial autophagic vacuoles (AVi) with double membranes, as indicated by the red squares. (**C**) Within the AVi and AVd, the Golgi apparatus (G) and degraded *R. buchneri* are visible, highlighted by the red arrows. A duplicating *R. buchneri* is observed in the lower right of the image. (**D**) Two degraded *R. buchneri* with double membranes are observed within the AVi, indicated by the red arrow.
