# Supplementary material for: The role of autophagy in tick-endosymbiont interactions: insights from Ixodes scapularis and Rickettsia buchneri
Source: Microbiol Spectr. 2023 Dec 1;12(1):e01086-23. doi: 10.1128/spectrum.01086-23 (PMC10783069; doi:10.1128/spectrum.01086-23)
Supplement: Table S1 — Primers in this study. [file spectrum.01086-23-s0008.docx]

| Gene name | Forward primer (5’-3’) | | Reverse primer (5’-3’) |  |
| --- | --- | --- | --- | --- |
| *Atg2* | CCCCTAACGGACAACAGCAT | ACGAATCGTCGTGTTGGTCA | |  |
| *Atg3* | TGACGCTGGAGCCTAATGTG | GGGGTGCGGTAGTAGTTGTC | |  |
| *Atg4A* | CTCTCGCCATTCATGTTGCG | ACTGTTTCAGCGCAAACGTC | |  |
| *Atg4B* | GCTGATGTCCGTCTGGAACA | AGATGGGAGCGTCCTTGTTG | |  |
| *Atg5* | GAGAAGGCTACGCTGGAGAC | TGAGTCACCACTGAGAACGC | |  |
| *Atg6* | GGCCAGCCTATCGGTAACTC | AGAGTCGTCGTTTGGGAACC | |  |
| *Atg7* | CGCCCTAGACGGAACCTTTT | GAGTTTGGGAGCCAGATGCT | |  |
| *Atg8A* | AGGAGCTTCAAGGAACGCAA | GCATGCCTGACAATGGACAC | |  |
| *Atg8B* | TGTTGCAGGTGATCGTGGAG | TTCTCGGGAGACAGGTGGAT | |  |
| *Atg8C* | CAGCATGTTGACGGTTTGGG | CGTCCGGTTAGCAATGCAAG | |  |
| *Atg11* | TGTCGGATACCTTTGGCACC | ACAGGTCCAAGCACGAAGAG | |  |
| *Atg13* | GAAGCTGTCTCCACACGACA | CCAGTCGGAGACCGTTGAAA | |  |
| *Atg16* | CACGACCGCACTCTCAAGAT | ATCTCGTTGGCACTGCTCTC | |  |
| *Atg101* | CATGAAGGATGCCAACCAGC | CAGGGACGAGAAGTTTCGCT | |  |
| *gapdh* | ATTGGAGACACCCACAGCAG | GACACGCTTCACTGGTCCTT | |  |
| 8ASiRNA | AAGCCTGCCACTGTTAGACAACCTGTCTC | AATTGTCTAACAGTGGCAGGCCCTGTCTC | | |
| Scambled-8ASiRNA  8CSiRNA  Scambled-  8CSiRNA | AAGACCTACATCGCGAAGTCTCCTGTCTC  AACCCTGTTCTTCATTGTCAACCTGTCTC  AAGCCCATTTACCGTTTCATTCCTGTCTC | AAAGACTTCGCGATGTAGGTCCCTGTCTC  AATTGACAATGAAGAACAGGGCCTGTCTC  AAAATGAAACGGTAAATGGGCCCTGTCTC | | |

Table S1: Primers in this study.
